# Concentration-Dependent Feeding Deterrence to 20-Hydroxyecdysone for Three Subterranean Termite Species (Blattodea: Rhinotermitidae)

**DOI:** 10.3390/insects12030218

**Published:** 2021-03-04

**Authors:** Lucas Carnohan, Sang-Bin Lee, Nan-Yao Su

**Affiliations:** 1 American Pest, 11820 West Market Place, Fulton, MD 20759, USA; lcarnohan@americanpest.net; 2 Department of Entomology and Nematology, Ft. Lauderdale Research and Education Center, University of Florida, 3205 College Avenue, Ft. Lauderdale, FL 33314, USA; nysu@ufl.edu

**Keywords:** termite bait, hyperecdysonism, *C. formosanus*, *C. gestroi*, *R. flavipes*

## Abstract

**Simple Summary:**

Subterranean termite colonies can be eliminated using baiting systems. However, for a given bait to be effective, the active ingredient must be lethal at concentrations that are also palatable to termites. The insect molting hormone, 20-hydroxyecdysone (20E), has potential for use in termite baits, but its palatability to termites has not been examined. The purpose of this study was to determine what concentrations of 20E, if any, cause termite workers to feed less readily. To test this, paper disks were treated with various concentrations of 20E. Groups of 1000 termites of three different species; the Formosan, the Asian and the Eastern subterranean termite; were placed in arenas. The termites had the option of following a path to feed on either a paper disk containing the 20E, or an untreated disk, and the amount of paper consumed was then compared. The results showed that the Asian subterranean termite had the least tolerance for the 20E, the Formosan subterranean termite had a reduced tolerance, and the presence of the 20E had no impact on the Eastern subterranean termite.

**Abstract:**

Effective active ingredients in toxicant bait formulations must be non-deterrent to insect feeding behavior at lethal concentrations. This study evaluated feeding deterrence for *Coptotermes formosanus* Shiraki, *C. gestroi* (Wasmann), and *Reticulitermes flavipes* (Kollar) when provided access to cellulose impregnated with various concentrations of the insect molting hormone, 20-hydroxyecdysone (20E). Termites were exposed to 20E concentrations of 200, 500, 1000 and 2000 ppm and to noviflumuron at 5000 ppm in a 24 h choice-test, and the mass of substrate consumption from treated and untreated media pads was compared for each treatment. 20E feeding deterrence was detected at 500, 1000 and 2000 ppm for *C. gestroi*, and at 2000 ppm for *C. formosanus*. No significant differences in consumption of treated and untreated substrate was detected at any concentration for *R. flavipes*. Potential methods for reducing deterrence are discussed.

## 1. Introduction

Subterranean termites cause significant damage to structures with global economic costs associated with termite treatment and prevention, and structural repairs estimated at USD 32 billion/year [1]. In the United States, the majority of structural damage is caused by the *Coptotermes* (Blattodea: Rhinotermitidae) and *Reticulitermes* (Blattodea: Rhinotermitidae) genera. To eliminate termite colonies and prevent expensive structural damage, the use of baiting systems is one viable option and has been a useful management tool in the US and throughout the rest of the world [1,2,3].

One group of insect growth regulators, the chitin synthesis inhibitors (CSIs), have proven effective as active ingredients (AIs) in baiting systems [4,5,6]. Subterranean termite colonies consuming CSI bait formulations are eliminated, providing area-wide structural protection for extended periods of time [2,3,7,8,9]. Successful termite baiting systems were only made possible by CSI’s unique characteristics. They are slow-acting and have dose-independent lethal times in treated subterranean termite populations [2,10]. Furthermore, the foraging tunnels of termite colonies weakened or eliminated using CSIs are likely to be invaded by neighboring colonies, which can lead to the elimination of additional colonies, thereby expanding the termite-free zone around a structure [11]. CSIs such as hexaflumuron have also been demonstrated in laboratory studies to have a broad efficacy range of 125 ppm through 4000 ppm [12]. At concentrations within this range, *Reticulitermes flavipes* (Kollar) and *Coptoterems formosanus* Shiraki did not show feeding deterrence, and 100% mortality was observed after a 9-week period [12]. The suitability of CSI baits for use in subterranean termite colony elimination has been reaffirmed many times over the past 20 years [3]. 

However, a major drawback of current termite baiting systems using CSIs is the length of time required for colony elimination. According to a summary of termite baiting over the past 20 years, the minimum and maximum average time required to eliminate a single termite colony using CSIs was 111 ± 14 and 190 ± 16 days, respectively [3]. For the elimination of all termites around a structure, the average minimum and maximum time was 105 ± 20, and 272 ± 54 days, respectively [3]. The length of time required for successful colony elimination using baiting systems consists of: bait interception time, bait toxicant acquisition time and lethal time that is related to the mode of action of CSIs [2,6,13]. Once foraging termites consume the CSI baits, it will be spread out within the colony through trophallaxis [8,14,15]. This chemical class interferes with the molting process, so it does not have any effects until termites initiate the molting process. It has been demonstrated that foraging populations of *C. formosanus* will molt two weeks after field collection [16], indicating that termites that have consumed a lethal dose of a CSI do not die until reaching their next molting cycle, at which point ecdysis cannot be successfully completed [17,18]. A recent study found that termites always return to the central nest prior to molting where reproductives, eggs and younger brood are located, and die near them if termites have ingested CSI baits [19].

In this regard, it may be possible to accelerate the molting process through inducing hyperecdysonism in termites, and this could speed up colony elimination using baiting systems. Hyperecdysonim can be induced through termite consumption of ecdysteroids and ecdysone agonists [13,20]. Previously, cellulose substrate containing ecdysone, 20-hydroxyecdysone (20E), and halofenozide were provided to *C. formosanus* and *R. flavipes* for 12 days and it caused significant mortality [13]. In a similar study, 20E was shown to have dose-independent characteristics similar to CSI’s but with a significantly faster rate of mortality [20]. The times required for 20E to have significant mortalities were about 13 days for *C. formosanus*, *C. gestroi* (Wasman), and *R. flavipes* [20]. Generally, higher concentrations of 20E caused higher mortalities, but the highest mortalities were achieved with a combination of noviflumuron and 20E [20]. 

For an AI to serve as an effective toxicant in a bait, termites cannot be deterred from ingesting it at lethal concentrations [21]. If a given species of termite is deterred from feeding on an AI at a concentration that is lower than the required concentration to cause mortality, then the compound will not be effective as a bait toxicant because termites cannot be force-fed in the field [5]. For example, hexaflumuron was shown to be a much better AI for use in baiting than lufenuron, because deterrence was not observed until 8000 and 4000 ppm for *C. formosanus* and *R. flavipes*, respectively [12]. On the other hand, termites exhibited feeding deterrence to lufenuron at concentrations as low as 1000 ppm for *C. formosanus*, and 50 ppm for *R. flavipes* [12]. 

Laboratory choice tests have been used to determine deterrence thresholds for potential bait toxicants [22,23]. In a choice test, it is important that the termites have an alternative food source that does not contain the AI of interest, as well as the ability to physically avoid the treatment compound [21]. Subterranean termite deterrence to a given AI can change over time [22,24]. In the case of a deterrent AI, it may be attributable to termites acquiring a sub-lethal dose and communicating the presence of the bait to the rest of the colony, perhaps through associative learning [25], or marking the bait with a warning pheromone. It has been shown that one of the most common immediate effects of ecdysteroid consumption in insects is a discontinuation of feeding behavior after 3–14 h [26]. 

Despite the promising characteristics of 20E for potential use in bait systems, its feeding deterrence thresholds are currently unknown for any economically important termite species. In this study, we examine feeding deterrence of 20E in *C. formosanus*, *C. gestroi*, and *R. flavipes* using a choice test in order to evaluate 20E as a bait toxicant for subterranean termites. 

## 2. Materials and Methods

### 2.1. Termites

Samples of *C. formosanus* were collected from field colonies in South Florida, using a modified version of a method described by Tamashiro et al. (1973) [27] and Su and Scheffrahn (1986) [28]. Samples of *C. gestroi* were obtained from 4 year-old laboratory reared colonies and contained total populations of ~30,000–50,000 individuals. *C. gestroi* colonies were contained in plastic boxes (Carlisle 30.5 × 45.7 × 15.2 cm^3^, 13 L Oklahoma City, OK, USA), and samples were extracted by placing damp rolls of corrugated cardboard into each colony box for several hours. Workers and soldiers would quickly move into the corrugated cardboard, which could then be removed and unrolled to extract the termites. *Reticulitermes flavipes* samples were collected from field colonies in Vero Beach, FL and from small colonies that were collected in Pompano, FL, USA and maintained in the laboratory.

### 2.2. Media Pad Preparation

Cellulose media pads (AP 10, 45 mm, Millipore Corp., Billerica, MA, USA) were placed in glass Petri dishes (90 mm diameter) and dried in an oven at 70 °C for at least 8 h before weighing to obtain the dry weights. Dried pads were treated with 1 mL of a methanol solution containing the AI of interest to yield the concentrations of 200, 500, 1000, and 2000 ppm (*w*/*w*) for 20E, 5000 ppm for noviflumuron, and an untreated control. The methanol was allowed to evaporate from the media pads for two hours before they were exposed to termites.

### 2.3. Choice Test

**Figure 1:**
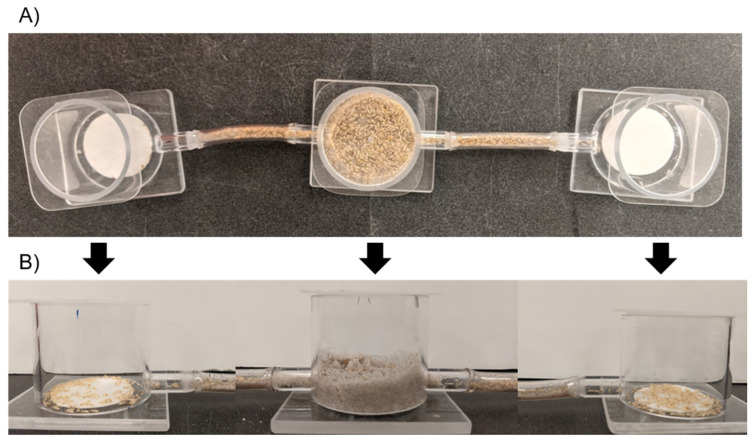
Choice assay with a central release chamber (center), and two side chambers; one with treated media pad and one with untreated media pad. Top (**A**) and side (**B**) view of the choice assay.

Three Plexiglas chambers (5.7 cm diam. × 4.8 cm high) were connected using two 5-cm pieces of flexible vinyl tubing (10 mm OD, 7 mm ID, Whatts, N. Andover, MA, USA) between chambers (Figure 1). Sand was washed three times with ethanol and sifted using 500 mm mesh. Then, moist sand (35 g/5 mL, sand/deionized water) was inserted into the central chamber as well as the connective tubing to provide the termites with a graspable building material. Media pads, one treated and one untreated, were placed in the left and right chambers, respectively, and moistened with deionized water.

Groups of 1000 termite workers (undifferentiated larvae of at least the 3rd instar) were counted and partitioned using a breath aspirator. To maintain the appropriate soldier ratio, 100 soldiers were added to each group for *C. formosanus* and *C. gestroi*, and 10 soldiers were added to *R. flavipes* groups [29]. Termites were introduced into the center chamber, and each unit was then covered with black plastic sheets to provide a dark environment and kept at 27.5 ± 1 °C and 80 ± 1% of relative humidity.

To determine whether termites display deterrence to 20E and/or noviflumuron that is not related to the moribund condition of intoxicated individuals, termites were exposed to the compounds for only 24 h. This time-period was selected based on preliminary trials, which revealed that termites exposed to 20E and noviflumuron for 24 h showed no signs of being moribund. After 24 h both media pads were removed from the unit and placed back in the oven set to 70 °C. After drying for at least 8 h, each media pad was gently brushed to remove any sand debris and reweighed to determine the amount consumed by termites during the 24 h exposure. The total consumption that occurred for both the treated and untreated media pads combined for each replicate was also calculated. 

A replicate consisted of six choice arenas; one for each treatment. The experiment was replicated at least four times for each species. For *C. gestroi* there were five control replicates, and four replicates for all other treatments. For *C. formosanus* there were exactly four replicates for each treatment. For *R. flavipes*, there were six 5000 ppm noviflumuron replicates, five replicates for the control, 200 ppm 20E, and 1000 ppm 20E, and four replicates for 500 ppm 20E and 2000 ppm 20E. For *C. formosanus* and *C. gestroi*, termites from the same colony were used for all six treatments for each replicate, with a different colony for each replicate. For *R. flavipes*, a variety of colonies were used, and due to small colony populations, some replicates contained multiple colonies.

### 2.4. Statistical Analysis

Differences in consumptions of media pads between treated and untreated control for each treatment were compared for each species by using a pairwise *t*-test. Total consumption (treated plus untreated media pads) were analyzed using an ANOVA to detect significant difference (α = 0.05) among treatments for each termite species. All analyses were performed with SPSS V20 [30]. All data were deposited in the supplementary materials (Supplementary file S1).

## 3. Results

For the three termite species tested, there were no significant differences among treatments for the total amount (treated plus untreated) of media pad consumed (Table 1). Termites showed similar levels of total consumption, regardless of the treatment. 

**Figure 2:**
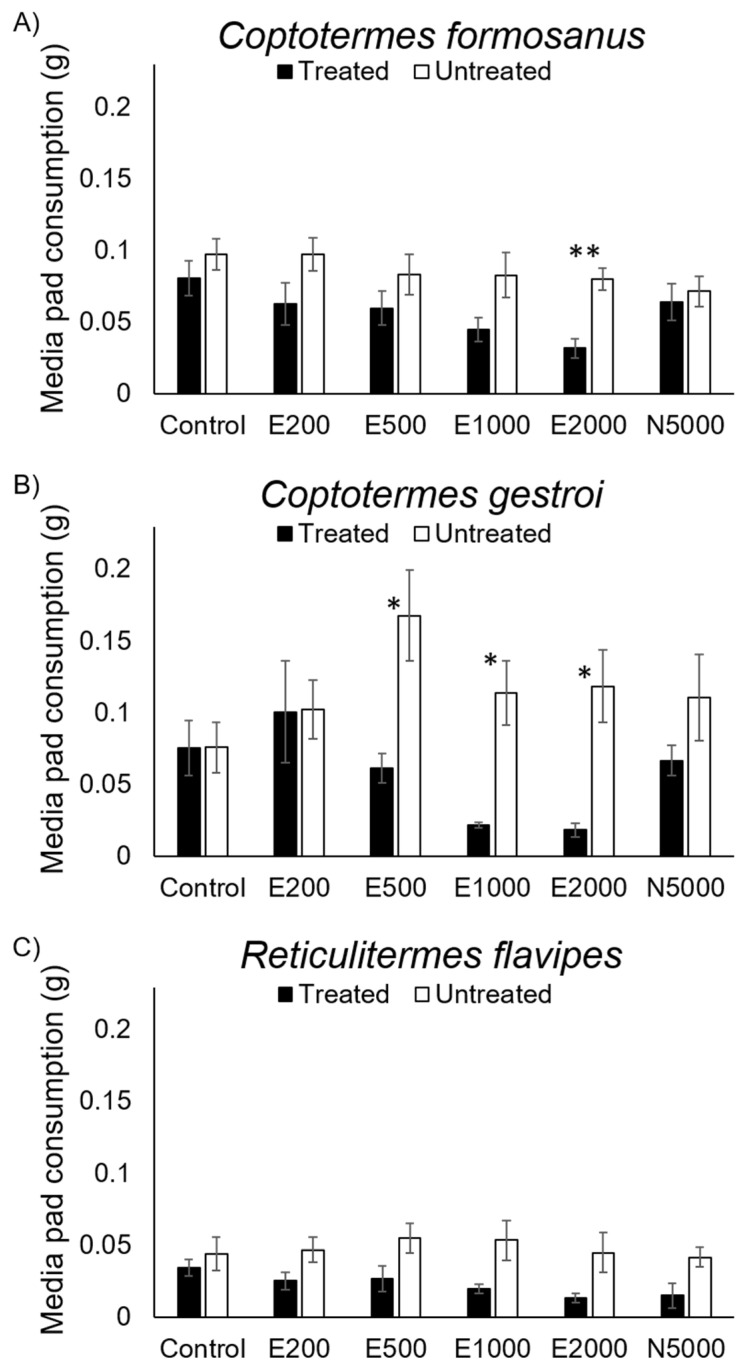
Average amount of media pad consumed from treated and untreated media pad in a 24-h choice test for *C. formosanus* (**A**), *C. gestroi* (**B**), and *R. flavipes* (**C**). E and N indicate 20E and noviflumuron respectively. Concentrations of 20E were 200, 500, 1000 and 2000 ppm, and of noviflumuron was 5000 ppm. Asterisk denote significant different consumption between treated and untreated media pads according to paired *t*-test (*: *p* < 0.05, **: *p* < 0.01).

*C. formosanus* consumed significantly less of the media pads treated with 20E at 2000 ppm than untreated media pads (t = −4.068; df = 6; *p* < 0.01) (Figure 2A), whereas *C. gestroi* consumed significantly less of the media pad treated with 20E at 500 (t = −2.760; df = 6; *p* = 0.03), 1000 (t = −3.530; df = 6; *p* < 0.05) and 2000 (t = −3.402; df = 6; *p* < 0.05) ppm than untreated media pad (Figure 2B). Consumption quantities of treated and untreated media pads by *R. flavipes* were not significantly different for any treatments (Figure 2C), and noviflumuron did not deter feeding at 5000 ppm for the three termite species tested (Figure 2). 

For all replicates, the observed visual termite activity was equal in all three chambers of the arena, though *R. flavipes* appeared to take longer to move out of the release chamber into the side chambers. Termites did not avoid the chambers containing the treated media pad, even for the treatments where significant differences in consumption were observed. 

## 4. Discussion

Baits containing CSIs are known to cause mortality in termites through disruption of the molting process [2,6,19]. Similarly, individuals that fed on 20E (100 and 1000 ppm) exhibited hyperecdysonism in *C. formosanus* and *R. flavipes*, and displayed dose-independent mortality while the mortality occurred in a shorter time period (≈12 days) than CSIs, suggesting 20E could be used to enhance the speed of termite baiting systems [13,20]. Previous experimental designs were no-choice tests, and the possibility of termite deterrence to 20E was not evaluated. For an AI to be effective in baiting systems, a lack of feeding deterrence at lethal concentrations is essential. In this study, we showed a lack of significant differences in total media pad consumption among treatments for all three termite species, indicating overall termite feeding activities during the 24 h test period were not adversely affected by the treatments (Table 1). If a certain chemical type/concentration had caused the termites to become moribund within the 24-h period, it was expected that the given treatment would show significantly less total consumption than the untreated control. 

Furthermore, an effective bait should not cause any feeding deterrence within the effective lethal concentration range [21,22]. In *R. flavipes,* no significant feeding deterrence was observed across different concentrations, and feeding deterrence of *C. formosanus* was recorded only at high concentration (2000 ppm) (Figure 2). However, *C. gestroi* showed significant feeding deterrence to a broad range of concentrations (i.e., 20E at 500, 1000 and 2000 ppm). These findings help explain why 20E induced lower overall mortality in *C. gestroi* than in *C. formosanus* in a previous study [20]. 

Additionally, higher mortalities were recorded and the lethal time was significantly reduced when 20E (≥1000 ppm) in combination with noviflumuron (2500 ppm) was fed to termites [20]. Combining results from this and previous studies, it is possible that 1000 ppm of 20E in combination with noviflumuron could provide both reduced time period for baiting and a lack of feeding deterrence in *C. formosanus* and *R. flavipes*. Mortalities of *C. formosanus*, and *R. flavipes* force-fed with lower concentrations (500 ppm) of 20E were significantly lower than those fed with higher (≥1000 ppm) concentrations, indicating that 20E must be used at 1000 ppm or higher to reliably induce hyperecdysonism of *C. formosanus* and *R. flavipes* [20]. 

In this study, all three termite species showed foraging activity in both of the choice chambers throughout the 24 h of exposure, yet there was clear consumption bias between the control and treatment media pad for higher concentrations of 20E for *C. gestroi* (≥500 ppm) and *C. formosanus* (2000 ppm). However, it is important to note that higher concentrations of 20E did not cause physical avoidance of the treated media pads. This brings up an interesting issue regarding the nature of the feeding deterrence. It is possible that the termites sampled the 20E-treated media pad, found it distasteful, and then discontinued feeding. In that scenario, it may be possible to mask the presence of 20E in the media pad by adding a phagostimulant. One possible candidate may be wood/litter decay fungal metabolites as subterranean termites showed preference on the extracts and displayed much higher tunneling activities on sawdust treated with such metabolite extracts [31]. Therefore, adding such phagostimulant to a bait matrix containing 20E even at higher concentrations may reduce the deterrence that was observed in this study.

Baits containing CSIs have provided a viable option for homeowners and the pest control industry because, unlike traditional liquid termiticide barrier systems, complete colony elimination is possible and bait systems are more sustainable and environmentally friendly as CSI baits employed 600-fold less insecticides than the liquid termiticide [2,32]. Furthermore, the liquid termiticide can kill only termites that contacted the treatment and few meters away from the treatment as it is fact acting and dose-dependent insecticide [33,34,35,36]. On the other hand, CSI baits are slow-acting and lethal time dose-independent insecticide, which allows to eliminate colonies of subterranean termite near the structures regardless of distances from the treatment [2,33,36,37]. Despite of baits’ sustainability and usefulness in termite control, the relatively long duration of time required to achieve colony elimination of subterranean termites is considered the least appealing aspect of baiting systems [2]. The use of 20E as the sole bait toxicant or in combination with noviflumuron could result in a shorter time to colony elimination than what is currently possible using baits formulated with CSIs alone [20]. However, the effects of 20E on whole colonies of subterranean termites is still unknown, as 20E has been tested only at the individual level thus far. Therefore, investigations at the colony level and field trials are warranted to further our understanding of how 20E might perform as an AI in subterranean termite baiting systems.

## Figures and Tables

**Table 1:** Total media pad consumption (mg ± SE) of *C. formosanus*, *C. gestroi* and *R. flavipes*. In each treatment, treated and untreated consumption combined and compared with one-way ANOVA (*p* < 0.05).

		Species		
		*C. formosanus*	*C. gestroi*	*R. flavipes*
Treatment	Control	178.38 ± 24.15	151.50 ± 39.58	78.82 ± 18.57
	20E 200 ppm	160.65 ± 27.59	203.03 ± 58.46	72.48 ± 16.12
	20E 500 ppm	143.13 ± 27.50	229.38 ± 38.27	81.90 ± 17.62
	20E 1000 ppm	127.80 ± 25.47	135.80 ± 27.86	73.16 ± 17.47
	20E 2000 ppm	111.98 ± 15.72	137.23 ± 27.52	58.60 ± 18.03
	Noviflumuron	135.88 ± 24.68	177.50 ± 46.61	50.98 ± 14.82
Statistics	Mean square	0.00248688	0.00594039	0.00073422
	F	0.99	0.79	0.52
	*p*	0.4520	0.5681	0.7577

## Data Availability

Data will be provided as a Supplementary Materials.

## Supplementary Materials

The following are available online at https://www.mdpi.com/2075-4450/12/3/218/s1, Supplementary file S1: Data_Concentration-dependent feeding deterrence to 20-hydroxyecdysone for three subterranean termite species.
